# Role of Genetic and Epigenetic Biomarkers in Treatment-Resistant Depression: A Literature Review

**DOI:** 10.3390/genes16121443

**Published:** 2025-12-02

**Authors:** Petra Sulić, Andrea Ražić Pavičić, Biljana Đapić Ivančić, Tamara Božina, Nada Božina, Maja Živković

**Affiliations:** 1 Clinical Department of Psychiatry and Psychological Medicine, University Hospital Centre Zagreb, 10000 Zagreb, Croatia; petrasulic@yahoo.com (P.S.); andrea.razic.pavicic@mef.hr (A.R.P.); 2 School of Medicine, University of Zagreb, 10000 Zagreb, Croatia; nada.bozina@mef.hr; 3 Department of Neurology, University Hospital Centre Zagreb, 10000 Zagreb, Croatia; biljana.djapic.ivancic@kbc-zagreb.hr; 4 Department of Medical Chemistry, Biochemistry and Clinical Chemistry, School of Medicine, University of Zagreb, 10000 Zagreb, Croatia; tamara.bozina@mef.hr

**Keywords:** treatment-resistant depression, major depressive disorder, pharmacogenetics, GWAS, *BDNF*, *NTRK2*, *GRIN2A*, *GRIN2B*, microRNA, epigenetics, ketamine, electroconvulsive therapy

## Abstract

Background: Treatment-resistant depression (TRD) affects up to 30–40% of patients with major depressive disorder and remains a major therapeutic challenge. Genetic and epigenetic factors are increasingly recognized as key contributors to both vulnerability and treatment response. Methods: We conducted a narrative review of studies published between 2021 and 2025, focusing exclusively on DNA- and RNA-based biomarkers of TRD. Twelve studies met the inclusion criteria, covering candidate gene analyses, genome-wide association studies (GWAS), neuroimaging–genetic approaches, and microRNA profiling. Results: Genetic investigations consistently implicate neuroplasticity-related genes (*BDNF*, *NTRK2*, *PTEN*, *SYN1*, *MAPK1*, and *GSK3B*) in the risk of TRD and its relapse. Variants in glutamatergic receptor genes (*GRIN2A*, *GRIN2B*, *GRIA2*, *GRIA3*) were predicted to result in a rapid and sustained response to ketamine. Genomic approaches further demonstrated that composite genetic panels outperform single-variant predictors. In parallel, microRNAs such as miR-1202, miR-16, miR-135, miR-124, miR-223, and miR-146a emerged as dynamic biomarkers of treatment response, particularly in cohorts treated with ketamine or electroconvulsive therapy. Conclusions: DNA- and RNA-based biomarkers provide promising avenues for improving the understanding and management of TRD. Their integration into clinical frameworks could support patient stratification, individualized treatment selection, and real-time monitoring of therapeutic efficacy. Future research should prioritize replication, methodological harmonization, and longitudinal validation to facilitate the translation of findings into precision psychiatry.

## 1. Introduction

### 1.1. Epidemiological and Clinical Context of TRD

Major depressive disorder (MDD) remains one of the leading causes of disability worldwide [1], affecting more than 300 million people, with lifetime prevalence estimates ranging between 12% and 20% depending on region [2,3]. MDD has been ranked as the third greatest cause of disease burden worldwide and is projected to rank first within the next decade [1,4]. It frequently follows a chronic and relapsing course, with recurrence observed in approximately 50% of patients after a first episode, 70% after a second, and up to 90% after a third [1,5]. Although standard antidepressants achieve remission in a subset of patients, up to 30–40% fail to respond to at least two adequate treatment trials, meeting criteria for TRD [6,7,8,9,10,11,12,13]. This subpopulation carries a disproportionate burden, including chronic course, economic costs, high relapse rates, and elevated suicide risk [14,15,16,17,18,19,20,21,22].

For decades, depression research was dominated by the monoaminergic hypothesis, which attributed pathophysiology to reduced serotonergic, noradrenergic, and dopaminergic signaling [23,24,25]. While this framework contributed to the development of SSRIs and SNRIs, its explanatory power is limited, as only one-third of patients achieve remission after first-line therapy, and effect sizes remain modest in TRD [26,27,28]. These shortcomings have prompted a paradigm shift toward alternative mechanisms, including neuroplasticity impairment [29,30,31,32,33].

### 1.2. Neuroplasticity as a Core Mechanism of Treatment Response

Emerging translational evidence has therefore shifted focus toward the cellular and molecular basis of antidepressant response, particularly mechanisms underlying neuroplasticity.

Neuroplasticity refers to the brain’s ability to reorganize its structure, synaptic connections, and functional networks in response to internal and external stimuli, including stress, learning, and pharmacological treatment. Impairment of neuroplasticity in depression manifests as reduced synaptogenesis, dendritic atrophy, and impaired neurogenesis in regions such as the hippocampus and prefrontal cortex, contributing to emotional dysregulation and cognitive dysfunction [29,30]. Genomic regulation of neuroplasticity involves several interconnected signaling cascades, most notably the BDNF–TrkB, MAPK–ERK, and mTOR pathways, which modulate synaptic remodeling, long-term potentiation, and neuronal survival [29,34,35]. Understanding the genetic and epigenetic variation within these pathways is therefore crucial for elucidating the biological mechanisms underlying treatment resistance and for developing targeted antidepressant strategies that restore neuroplasticity [36].

### 1.3. Pharmacogenomic Foundations and Genetic Variability

This molecular understanding has been paralleled by an expanding body of pharmacogenomic research.

Over the past decade, prior to the period analyzed in this review, several pharmacogenomic studies established the biological context for understanding TRD. Early investigations highlighted the role of genetic variability within monoaminergic, neurotrophic, and stress-response pathways in determining the likelihood of achieving remission with antidepressant therapy. Among the best-studied examples are the *BDNF* Val66Met polymorphism, which has been repeatedly associated with differences in neuroplasticity and hippocampal integrity, and *COMT* Val158Met and *SLC6A4* 5-HTTLPR variants, which modulate dopaminergic and serotonergic neurotransmission and have been linked to heterogeneous treatment outcomes [37,38].

Further evidence connected stress-regulatory mechanisms to treatment response, with *FKBP5* and *NR3C1* variants shown to alter hypothalamic–pituitary–adrenal (HPA) axis activity and vulnerability to stress-related depressive relapse [39,40]. Other genes, such as *CACNA1C*, *ANK3*, and *CLOCK*, involved in calcium signaling and circadian rhythm regulation, have been proposed to contribute to neuronal excitability and antidepressant sensitivity [41].

Meta-analyses and genome-wide studies from this period consistently suggested that TRD is not explained by a single genetic variant, but rather by the interaction of multiple molecular systems that converge on synaptic plasticity, glutamatergic neurotransmission, and inflammatory signaling [42,43]. These earlier insights formed the conceptual and methodological basis for the recent multi-omic and integrative studies published after 2021, which further explore how DNA and RNA biomarkers jointly shape therapeutic outcomes in TRD.

### 1.4. Neurotrophic and Synaptic Gene Networks

Genomic research provides mounting evidence for neuroplasticity-related pathways as determinants of treatment outcome.

Candidate gene analyses associate polymorphisms in *BDNF*, *NTRK2*, *PTEN*, *SYN1*, *MAPK1*, and *GSK3B* with TRD and relapse [44]. Neuroimaging–genetic studies link *NTRK2* variants with reduced hippocampal volume, a structural correlate of poor antidepressant response [45]. Broader glutamatergic contributions have also been documented, with *GRIN2B* polymorphisms linked to TRD risk, suicidality, and anterior cingulate glutamate levels [46]. Genome-wide approaches identify variants in *BDNF*, *NTRK2*, and *GRIN2A* that predict rapid and sustained ketamine effects, alongside associations with serum ketamine/norketamine levels [47]. Large-scale, multi-center analyses from the Predictors Consortium further validated these associations, demonstrating consistent signals in BDNF–TrkB and glutamatergic pathways across heterogeneous cohorts [48].

Among these findings, particular attention has been given to genes directly mediating neurotrophic signaling cascades.

Several key genes within neuroplasticity-related signaling cascades have been consistently implicated in treatment response and relapse vulnerability in major depressive disorder. *BDNF* encodes brain-derived neurotrophic factor, a central regulator of neuronal growth, synaptic strengthening, and activity-dependent plasticity. Its receptor, NTRK2 (TrkB), activates downstream signaling through MAPK1, PI3K–Akt, and mTOR pathways, all of which promote neuronal survival and dendritic remodeling. Reduced BDNF–TrkB activity has been repeatedly associated with chronic stress exposure, smaller hippocampal volume, and poor antidepressant response [36,49]. PTEN functions as a critical negative regulator of serotonergic neuroplasticity, where its overexpression diminishes axonal growth and synaptic density in 5-HT neurons, leading to depressive-like phenotypes, whereas its suppression enhances synaptic remodeling and resilience to stress-induced behavioral deficits [50]. Recent findings suggest that epigenetic mechanisms may also modulate Synapsin expression in mood disorders. DNA hypomethylation of SYN2 CpG islands has been associated with increased gene expression in the prefrontal cortex of patients with MDD and bipolar disorder, linking altered methylation at Synapsin loci to disrupted synaptic regulation in affective illness [51]. MAPK1 (ERK2) functions as a key effector of activity-dependent synaptic plasticity, translating external stimuli into long-term neuronal adaptations. Meanwhile, GSK3B participates in intracellular signaling that controls apoptosis and cytoskeletal dynamics. Dysregulated GSK3B activity has been associated with chronic inflammation, impaired antidepressant efficacy, and susceptibility to relapse after remission [52,53]. Collectively, these genes interact within a common neurotrophic–glutamatergic framework that determines the capacity for neuronal remodeling and sustained therapeutic response in TRD [34,54].

### 1.5. Glutamatergic Dysregulation and Synaptic Signaling

Given the intimate crosstalk between neurotrophic and glutamatergic pathways, disturbances in glutamate signaling have also gained increasing attention as core features of TRD pathophysiology.

The glutamatergic system has emerged as a central mechanism in the neurobiology of treatment-resistant depression. Glutamate, the major excitatory neurotransmitter in the central nervous system, regulates synaptic plasticity, learning, and emotional processing. Chronic stress, excessive glutamate release, or altered receptor composition can lead to excitotoxicity, dendritic atrophy, and impaired synaptic communication—core features of TRD pathology [34,55].

Among glutamatergic receptors, N-methyl-D-aspartate (NMDA) and α-amino-3-hydroxy-5-methyl-4-isoxazolepropionic acid (AMPA) play complementary roles: NMDA receptors mediate calcium-dependent signaling and long-term potentiation, whereas AMPA receptors facilitate fast excitatory neurotransmission. Dysregulation of NMDA and AMPA receptor balance disrupts neuronal connectivity and stress resilience. Antidepressant interventions targeting these receptors—particularly NMDA antagonists such as ketamine—restore excitatory balance, enhance AMPA throughput, and promote neuroplasticity [30,35].

### 1.6. Ketamine and Rapid-Acting Antidepressant Mechanisms

Ketamine has become a cornerstone in understanding the neurobiology of treatment-resistant depression because of its ability to produce rapid and robust antidepressant effects in patients who fail to respond to conventional monoaminergic drugs. Unlike selective serotonin reuptake inhibitors (SSRIs), which require several weeks to induce clinical improvement, subanesthetic doses of ketamine act within hours by modulating glutamatergic neurotransmission and enhancing synaptic connectivity [30,56].

Mechanistically, ketamine blocks NMDA receptors on GABAergic interneurons, resulting in the transient disinhibition of glutamatergic pyramidal neurons and an increase in extracellular glutamate. This leads to enhanced AMPA receptor activation and downstream stimulation of the BDNF–mTOR signaling cascade, which promotes synaptogenesis, spine formation, and restoration of functional neural networks in mood-related circuits [29,54].

These mechanisms explain ketamine’s unique capacity to reverse neuroplastic deficits underlying chronic and treatment-resistant depression.

### 1.7. Epigenetic and RNA-Based Regulation of Neuroplasticity

Beyond pharmacological modulation, accumulating evidence highlights the importance of transcriptional and post-transcriptional mechanisms in sustaining neuroplastic adaptations.

In parallel with genetic studies, recent research has focused on RNA-based mechanisms, particularly microRNAs (miRNAs), which fine-tune gene expression at the post-transcriptional level. miRNAs are short, non-coding RNA molecules that bind to target mRNAs and suppress their translation, thereby regulating neuronal development, stress response, and synaptic plasticity. Altered miRNA expression profiles have been consistently observed in patients with major depressive disorder and treatment-resistant depression, reflecting dysregulation of neuroplastic and inflammatory pathways [57,58]. Specific miRNAs, such as miR-16, miR-1202, miR-135, miR-124, and miR-146a, modulate serotonergic signaling, BDNF expression, and immune processes. In contrast, the miR-223 and miR-29 families have been linked to treatment response following electroconvulsive therapy and ketamine exposure [59,60]. Collectively, these findings position miRNAs as both mechanistic mediators and promising circulating biomarkers for monitoring the efficacy of antidepressants in TRD. Electroconvulsive therapy (ECT)-focused studies report downregulation of inflammatory miRNAs such as miR-223 and miR-146a in responders, with non-responders showing no changes [61,62]. Importantly, both ketamine and ECT converge on overlapping networks, including the miR-29 family and the miR-132/212 cluster, which regulate apoptotic signaling, neuroprotection, and hippocampal neurogenesis [63].

### 1.8. Rationale and Objectives of the Review

To date, systematic reviews and meta-analyses have investigated selected genetic and RNA biomarkers of treatment-resistant depression, often focusing on glutamatergic genes, neurotrophic pathways, and microRNAs. However, no prior review has comprehensively synthesized both DNA and RNA biomarkers across pharmacological (ketamine, esketamine) and somatic (ECT, TMS) interventions. This narrative review addresses this gap by integrating findings from 12 recent studies published between 2021 and 2025, highlighting candidate genes, GWAS findings, and microRNA regulation as promising predictors of treatment outcomes in TRD.

## 2. Materials and Methods

This narrative review was conducted by systematically searching the electronic databases PubMed (https://pubmed.ncbi.nlm.nih.gov (accessed on 15 June 2025) and Scopus (https://www.scopus.com (accessed on 15 June 2025). The search covered the period from 1 January 2021 to 1 June 2025, corresponding to the publication years of the included studies. The search strategy combined terms related to depression, treatment resistance, pharmacogenetics, and molecular biomarkers: “treatment-resistant depression,” “major depressive disorder,” “pharmacogenomics,” “genetics,” “RNA,” “microRNA,” “epigenetics,” “ketamine,” “esketamine,” “ECT,” and “TMS.”

Inclusion criteria were

1. Peer-reviewed original research articles or reviews published in English.
2. Human studies or translational studies with direct relevance to major depressive disorder or treatment-resistant depression.
3. Investigations of DNA variants (candidate gene studies, GWAS, multi-omic models with genetic focus) or RNA-based biomarkers (gene expression, microRNAs).

Exclusion criteria included conference abstracts, case reports, animal-only studies without a translational link, and studies that focused solely on proteomic or metabolomic biomarkers.

A total of 12 studies fulfilled these criteria. The characteristics of the studies (authors, year, methodology, and biomarker domain) are summarized in Table 1. These included candidate gene analyses, neuroimaging–genetic association studies, GWAS of ketamine response, systematic reviews of glutamatergic and neurotrophic genes, and multiple investigations of microRNA expression in TRD and ECT cohorts.

Limitations of this approach include its non-systematic design, restriction to English-language studies, and the heterogeneity of methods and outcome measures, which reduce comparability across studies.

## 3. Results

### 3.1. Genetic Predictors of TRD

Santos et al. [44] investigated polymorphisms in neuroplasticity-related genes in 80 patients with MDD treated under the Texas Medication Algorithm over 27 months. Three independent signals were identified for TRD: *PTEN* rs12569998 (TG genotype/G allele ≈ fourfold higher risk), *SYN1* rs1142636 (GG genotype ≈ sixfold, G allele ≈ threefold higher risk), and *BDNF* rs6265 (CT genotype ≈ 3.2-fold higher risk), where the T allele corresponds to the Met (variant) form and the C allele to the Val (wild-type) form. For relapse, two loci emerged: *MAPK1* rs6928, where C-allele carriers showed reduced relapse risk and longer time-to-relapse compared with GG homozygotes, and *GSK3B* rs6438552, where G-allele carriers had a substantially higher relapse risk; in this context, the C allele of *MAPK1* represents the protective variant, while the G allele of *GSK3B* denotes the risk variant as reported by Santos et al. [44]. Gene-set analyses converged on synaptic and glutamatergic signaling, implicating glutamate receptor activity and ionotropic receptor complexes as central pathways, consistent with the functional roles of *BDNF*, *MAPK1*, and *GSK3B* in regulating neuronal excitability and synaptic plasticity that underlie antidepressant response and relapse vulnerability in TRD.

Saez et al. [46] synthesized evidence implicating the glutamatergic system—particularly NMDA and AMPA receptor genes—in the pathogenesis of TRD and in the antidepressant response to ketamine and esketamine. *GRIN2B* polymorphisms (rs1805502, rs1806201, rs890) were identified as risk factors for TRD, suicidality, and reduced anterior cingulate glutamate, while *GRIN2A* rs16966731 was associated with rapid and sustained ketamine response. In preclinical models, deletion of the NR2B subunit in GABAergic interneurons abolished ketamine’s antidepressant-like effects, highlighting the necessity of NMDA receptor integrity for therapeutic efficacy. NR2B refers to the GluN2B subunit of the NMDA receptor, encoded by the *GRIN2B* gene, which mediates calcium-dependent synaptic signaling. This subunit plays a crucial role in ketamine’s mechanism of action, as its transient blockade enhances glutamate release, facilitates AMPA receptor activation, and triggers downstream BDNF–mTOR signaling leading to synaptic potentiation and rapid antidepressant effects. On the other hand, *GRIA2* and *GRIA3* variants, encoding AMPA receptor subunits, were correlated with earlier onset of MDD and suicidal ideation, and AMPA receptor activation was shown to be essential for sustaining ketamine’s antidepressant effects. Collectively, these findings suggest that coordinated NMDA inhibition and AMPA potentiation regulate synaptic plasticity, BDNF release, and glutamate signaling that underpin both TRD vulnerability and the mechanism of ketamine and esketamine response.

Paolini et al. [45] examined 121 MDD inpatients using structural MRI and targeted genotyping, identifying an *NTRK2* variant (rs1948308) with over-dominant effects. Heterozygotes exhibited smaller bilateral hippocampal volumes and higher odds of TRD compared with homozygotes. *NTRK2* encodes the TrkB receptor, the high-affinity binding site for BDNF, which mediates neurotrophic signaling essential for neuronal survival, synaptic remodeling, and hippocampal neurogenesis. Variations in *NTRK2* can alter receptor activation or downstream signaling through the *PI3K–Akt* and *MAPK–mTOR* pathways, both of which are crucial for maintaining hippocampal integrity and mediating antidepressant responses. The mediation analysis performed by Paolini et al. showed that hippocampal volume partially accounted for the association between rs1948308 and TRD, suggesting that reduced TrkB-mediated plasticity contributes to smaller hippocampal volume and higher treatment resistance. The persistence of a significant direct effect, however, suggests that additional molecular mechanisms, independent of structural brain changes, may also play a role. No associations were found for *BDNF* variants, including the *Val66Met* polymorphism.

Chen et al. [47] performed a candidate gene-based GWAS in 65 TRD patients treated with low-dose ketamine. Predictors of rapid (≤240 min) and sustained (up to 14 days) response included *BDNF* rs2049048, multiple *NTRK2* variants (rs10217777, rs10868590, rs77918527), and NMDAR subunits such as GRIN2A, GRIN2B, GRIN2C, and GRIN3A. Pharmacogenomic analyses linked *GRIN2A/2B* polymorphisms to circulating ketamine/norketamine levels and *NTRK2* variants to ketamine plasma concentrations at 80–120 min. Pathway enrichment confirmed glutamatergic signaling and activity-dependent plasticity as central mechanisms.

Kang et al. [65] analyzed genetic predictors of ketamine response in patients with TRD, reporting novel associations in genes regulating synaptic vesicle trafficking (*SYNGR1*, *VAMP2*) and immune regulation (*IL6R*, *TNFAIP3*). Enrichment analyses highlighted pathways related to synapse organization, neurotransmitter release, and cytokine signaling. Gene–gene interaction models suggest that inflammatory gene variants modulate ketamine efficacy through their effects on glutamatergic neurotransmission, indicating that combined synaptic–immune signatures may better stratify responders.

Zelada et al. [48] conducted a systematic review integrating genomic, transcriptomic, and protein-level findings to assess the predictive value of brain-derived neurotrophic factor (BDNF) in treatment response across MDD and TRD. The authors summarized evidence showing that both peripheral BDNF levels and genetic variation in *BDNF* and *NTRK2* influence antidepressant outcomes, including response to ketamine. Higher post-treatment BDNF concentrations and preserved BDNF–TrkB (NTRK2) signaling were consistently associated with greater symptom improvement, whereas lower BDNF activity correlated with treatment resistance. The review concluded that combining neurotrophic and glutamatergic markers—such as *BDNF*, *NTRK2*, and *GRIN2A/2B*—may enhance the precision of predictive models in TRD and support the future development of multi-omic frameworks in personalized psychiatry.

Franklin et al. [64] systematically reviewed genetic studies examining predictors of treatment response across ECT, TMS, ketamine, and esketamine. Candidate gene investigations most frequently evaluated variants in *BDNF* and *COMT*, with mixed and largely non-replicated associations reported for somatic treatments. For ketamine and esketamine, earlier reports had linked the *BDNF* Val66Met polymorphism to synaptic plasticity and treatment response; however, subsequent studies did not confirm these consistent effects. Genome-wide association studies remain underpowered, though several novel signals have been reported, including variants in genes related to immune regulation and synaptic function. Extensive registry-based studies of ECT demonstrated that polygenic risk scores for depression predicted poorer outcomes, while higher polygenic risk for bipolar disorder predicted better response. Overall, the review concluded that no single genetic variant has yet emerged as a reliable predictor of treatment outcomes.

Results are summarized in Table 2.

### 3.2. Epigenetic Regulation in TRD

Cătană et al. [60] reviewed the role of microRNAs (miRNAs) as biomarkers for treatment-resistant depression, highlighting their ability to regulate genes associated with immune responses, synaptic plasticity, and stress signaling. Candidate markers included miR-30a, miR-133b, miR-16, let-7 family, and drug-specific changes such as miR-1202 (citalopram) and miR-146a-5p (duloxetine). Several miRNAs normalized following successful treatment, suggesting their use as dynamic blood-based markers for monitoring antidepressant response.

Kaurani et al. [61] investigated miRNA expression in patients undergoing ECT and identified differential regulation of miR-146a, miR-223, and miR-126. These were linked to immune system modulation and neuronal plasticity pathways, supporting their role as biomarkers of ECT response.

Galbiati et al. [62] measured plasma miRNA levels before and after ECT in TRD patients, finding that miR-223-3p and miR-146a-5p decreased significantly in responders, while non-responders showed no significant changes. This suggests that these inflammatory miRNAs track therapeutic efficacy and could guide stratification for somatic treatments.

Cai et al. [33] conducted a systematic review of the involvement of miRNA in MDD and TRD. They highlighted consistent evidence for miR-1202, miR-16, miR-135, miR-124, and miR-146a as central regulators of synaptic plasticity, serotonergic signaling, and neuroinflammatory responses. The review proposed that these conserved miRNAs could form the foundation of standardized biomarker panels.

Statharakos et al. [63] examined epigenetic changes associated with somatic therapies, particularly ECT and ketamine, focusing on circulating miRNAs as biomarkers. They identified overlapping regulation of miR-29 family, miR-132, and miR-212, all of which are linked to synaptic plasticity, stress response, and neuronal survival. Notably, both ECT and ketamine were found to modulate miR-29a and miR-29c, which influence apoptotic and neuroprotective pathways, suggesting a shared mechanism of resilience induction. Furthermore, alterations in the miR-132/212 cluster were associated with improved cognitive performance and hippocampal neurogenesis following treatment. While the study emphasized the promise of miRNAs as cross-modality biomarkers, the authors highlighted several limitations, including small sample sizes, heterogeneous patient cohorts, and a lack of longitudinal validation. Nonetheless, their findings support the hypothesis that ECT and ketamine converge on common miRNA-mediated regulatory networks involved in antidepressant efficacy.

Results are summarized in Table 3.

## 4. Discussion

### 4.1. Genetic Predictors and Convergent Signaling

The strongest and most consistent genetic findings converge on BDNF–TrkB signaling and glutamatergic receptor biology. Candidate gene studies have identified polymorphisms in *BDNF*, *NTRK2*, *PTEN*, and *SYN1* as risk factors for TRD, while *MAPK1* and *GSK3B* variants predicted relapse [44]. These findings underscore the contribution of intracellular cascades—particularly Akt, ERK, and mTOR—that mediate synaptic plasticity, long-term potentiation, and dendritic remodeling. Neuroimaging–genetic work extends this evidence, with *NTRK2* rs1948308 carriers exhibiting smaller hippocampal volumes and increased resistance; mediation analyses confirm hippocampal atrophy as a biological pathway linking genotype to clinical outcome [45].

Genome-wide association studies have further strengthened these associations. *BDNF* rs2049048 and *NTRK2* rs10217777 predicted rapid and sustained ketamine effects, while *GRIN2A* and *GRIN2B* variants were associated with both clinical outcomes and ketamine/norketamine pharmacokinetics [47]. *GRIN2B* polymorphisms have also been tied to suicidality, stress-related vulnerability, and anterior cingulate glutamate concentrations, while *AMPAR* variants (*GRIA2/3*) contribute to disease onset and suicidality [46]. Integrative reviews suggest that these glutamatergic loci are relevant across modalities, including ECT and TMS, implying common neurobiological substrates across somatic and pharmacological treatments [64].

Beyond these core pathways, novel associations have been reported in calcium channel genes (*CACNA1C*), circadian regulators (*CLOCK*, *ARNTL*), and immune modulators (*IL6R*, *TNFAIP3*) [65]. Zelada et al. demonstrated that composite biomarker panels integrating glutamatergic, neurotrophic, and immune loci outperform single-variant models [48]. Together, these findings emphasize that TRD has a polygenic architecture, where converging synaptic, circadian, and immune pathways shape treatment trajectories.

### 4.2. Molecular Mechanisms and Signaling Networks

To integrate the genetic and molecular levels, recent research has increasingly focused on intracellular signaling and pathway-level disruptions that bridge genotype with neuroplastic and clinical outcomes.

**Figure 1:**
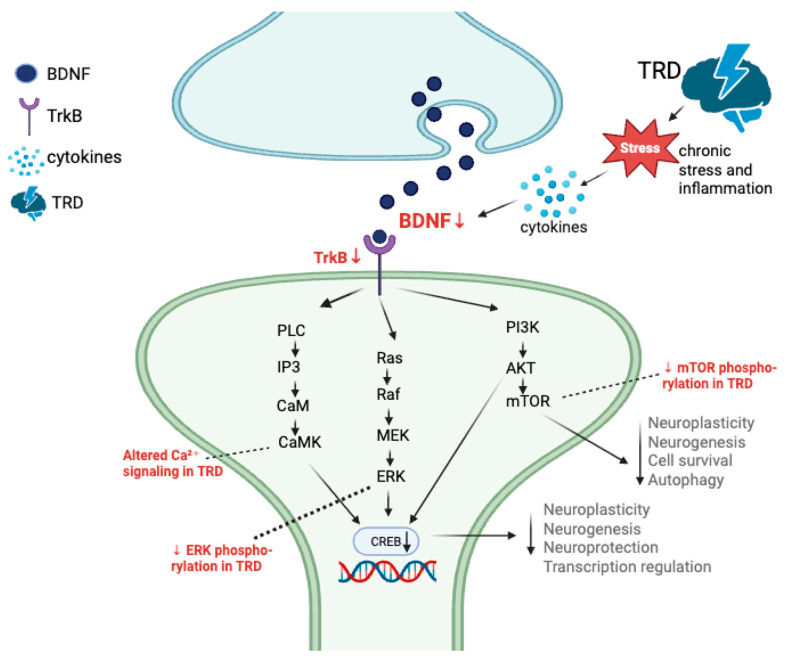
The BDNF–TrkB–MAPK/ERK–mTOR signaling axis in treatment-resistant depression Under physiological conditions, BDNF binds to its receptor TrkB, activating three major intracellular signaling cascades: (i) the MAPK/ERK pathway, which promotes CREB-dependent transcription of neuroplasticity-related genes; (ii) the PI3K–AKT–mTOR cascade, which regulates neuronal growth, survival, and synaptic protein synthesis; and (iii) the PLCγ–CaMK–CREB route, which controls Ca^2+^-dependent synaptic remodeling. In TRD, chronic stress, inflammation, and elevated glucocorticoids down-regulate expression and reduce TrkB phosphorylation, resulting in diminished ERK and mTOR activation, impaired protein synthesis, and synaptic atrophy. AKT, protein kinase B; BDNF, brain-derived neurotrophic factor; CaM, calmodulin; CaMK, calcium/calmodulin-dependent protein kinase; CREB, cAMP response element-binding protein; ERK, extracellular signal-regulated kinase; IP3, inositol 1,4,5-trisphosphate; MAPK/ERK, mitogen-activated protein kinase/extracellular signal-regulated kinase pathway; MEK, mitogen-activated protein kinase kinase; mTOR, mechanistic target of rapamycin; PI3K, phosphoinositide 3-kinase; PLC, phospholipase C; RAF, rapidly accelerated fibrosarcoma kinase; RAS, Ras GTPase protein; TrkB, tropomyosin receptor kinase B.

**Figure 2:**
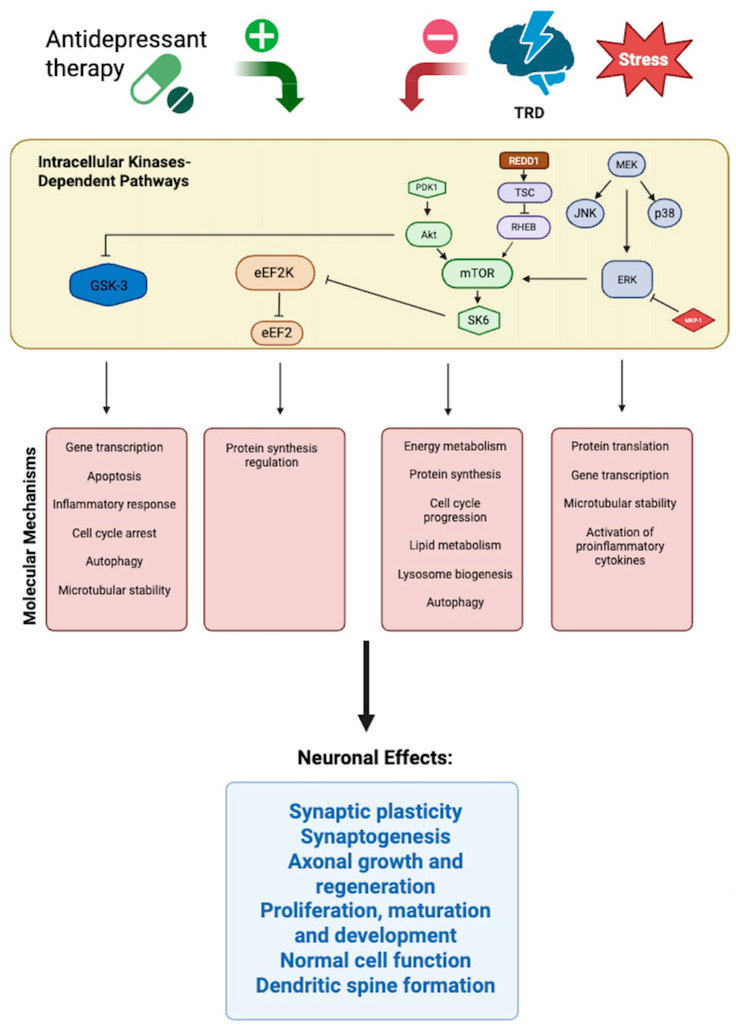
Kinase-centered intracellular networks as therapeutic targets in TRD Schematic representation of intracellular kinase-dependent signaling pathways involved in neuroplastic regulation and antidepressant response. Under physiological conditions, antidepressant therapy activates kinase cascades including Akt–mTOR, MAPK/ERK, and eEF2K–eEF2, which promote synaptic protein synthesis, energy metabolism, and neuronal survival. In contrast, chronic stress and TRD are associated with inhibition of mTOR and ERK signaling, activation of GSK-3, and increased REDD1 and TSC expression, leading to impaired neuroplasticity, synaptic dysfunction, and cellular atrophy. Pharmacological activation of mTOR or inhibition of GSK-3 reverses these maladaptive changes, restoring dendritic spine formation and synaptogenesis. Molecular processes regulated by these pathways include gene transcription, apoptosis, autophagy, cell-cycle progression, protein synthesis, and inflammatory response. Collectively, these kinase networks integrate metabolic, structural, and transcriptional mechanisms that converge on neuronal plasticity, regeneration, and resilience in TRD. Akt, protein kinase B; eEF2, eukaryotic elongation factor 2; eEF2K, eukaryotic elongation factor 2 kinase; ERK, extracellular signal-regulated kinase; GSK-3, glycogen synthase kinase 3; JNK, c-Jun N-terminal kinase; MEK, mitogen-activated protein kinase kinase; MKP-1, mitogen-activated protein kinase phosphatase-1; mTOR, mechanistic target of rapamycin; PDK1, 3-phosphoinositide-dependent protein kinase-1; p38, p38 mitogen-activated protein kinase; REDD1, regulated in development and DNA damage response 1; RHEB, Ras homolog enriched in brain; SK6, ribosomal protein S6 kinase beta-1; TSC, tuberous sclerosis complex.

At the molecular–mechanistic level, TRD reflects disruption across four interconnected signaling domains: (i) neurotrophic BDNF–TrkB–MAPK/ERK–mTOR pathways, (ii) glutamatergic NMDA/AMPA receptor balance, (iii) kinase-regulated intracellular cascades controlling synaptic remodeling, and (iv) epigenetic and post-transcriptional regulation. As visualized in Figure 1 and Figure 2, these systems interact to determine neuroplastic capacity, stress resilience, and antidepressant response.

Kinase pathways such as MAPK, mTOR, GSK3, and eEF2K regulate core molecular processes—including gene transcription, protein synthesis, autophagy, metabolism, and cell-cycle progression—that are essential for neuronal plasticity and survival [66]. Stress-related inhibition of these kinase-driven mechanisms disrupts molecular homeostasis within mood-regulating neurocircuits, leading to neuronal dysfunction and behavioral deficits characteristic of depressive states. Conversely, antidepressant-related activation of these kinase pathways—through agents such as ketamine, lithium, or mTOR modulators—restores synaptic signaling, promotes neurogenesis, and supports sustained remission (Figure 2) [34,52,54].

Kinases function as central molecular switches within these cascades. Analyses of the human kinome and translational psychiatry studies [67,68,69] identify MAPK/ERK (MAPK1/3), AKT–mTOR, and GSK3B as key druggable nodes. Reduced MAPK/mTOR activity and GSK3B overactivation impair neurotrophic signaling and dendritic complexity, whereas pharmacological modulation—mTOR activation or GSK3B inhibition—enhances synaptogenesis and antidepressant efficacy.

Post-translational modifications (PTMs) further refine these networks. Abnormal phosphorylation of MAPK1, CREB, and GSK3B disrupts BDNF transcription and synaptic remodeling [70]. Likewise, impaired hypusination of eIF5A—a unique lysine modification catalyzed by deoxyhypusine synthase (DHPS) and deoxyhypusine hydroxylase (DOHH)—compromises neuronal protein synthesis and viability, leading to dendritic atrophy and neurodevelopmental deficits [71,72]. These PTM-dependent processes represent an additional regulatory layer linking translational control to neuronal plasticity and may be therapeutically targeted in TRD.

Mechanistic overlap with neurodegenerative and epileptogenic pathways has also been proposed. TRD exhibits mechanistic overlap with neurodegenerative and epileptogenic pathways, particularly those involving glutamatergic dysregulation. In epilepsy, Eid et al. [73] demonstrated that increased expression of phosphate-activated glutaminase in hippocampal neurons, together with impaired astrocytic glutamate–glutamine cycling, promotes sustained excitotoxicity and hippocampal atrophy in mesial temporal lobe epilepsy. Analogous processes—excessive Ca^2+^ influx, mitochondrial dysfunction, and oxidative stress—have been implicated in the neuroprogressive course of TRD, suggesting a shared glutamate-driven neurotoxic continuum across these conditions. Complementary molecular evidence from Kar [74] further supports this convergence, highlighting excitotoxicity, kinase-dependent signaling, and metabolic stress as tractable therapeutic targets common to both epilepsy and treatment-resistant depression.

### 4.3. Epigenetic and RNA-Based Modulation of Neuroplasticity

Epigenetic regulation through miRNAs provides additional insight into treatment outcomes. Multiple miRNAs, including miR-30a, miR-133b, miR-16, miR-1202, and the let-7 family, are consistently dysregulated in TRD and show normalization with successful treatment [33,60].

ECT-focused studies have identified inflammatory miRNAs (miR-223, miR-146a) as markers of response, with responders showing downregulation and non-responders showing no significant changes [61,62]. These results indicate that blood-based miRNAs may serve as dynamic biomarkers of treatment engagement.

Systematic reviews confirm reproducibility for miR-1202, miR-16, miR-135, and miR-124, implicating them in serotonergic signaling, synaptic plasticity, and immune regulation [33]. Importantly, both ketamine and ECT converge on overlapping miRNA networks, specifically the miR-29 family (apoptosis and neuroprotection) and the miR-132/212 cluster (hippocampal neurogenesis and cognition) [63]. This suggests a shared mechanism across treatments where miRNAs act as both biomarkers and mechanistic mediators of antidepressant efficacy.

Beyond microRNAs, long noncoding RNAs (lncRNAs) have emerged as important regulators of neuroplasticity and stress response. LncRNAs interact with chromatin modifiers and splicing factors to fine-tune gene expression networks involved in neuronal differentiation and synaptic remodeling [75]. Their dysregulation has been linked to altered BDNF–TrkB signaling, aberrant neurotransmitter receptor splicing, and maladaptive stress responses—all processes relevant to treatment resistance in depression. Incorporating lncRNA profiles alongside miRNA and DNA variants may therefore enhance multi-omic precision approaches in TRD.

### 4.4. Translational and Clinical Implications

The convergence of genetic, molecular, and epigenetic findings offers a unified framework for biomarker-guided interventions in TRD.

1. Patient stratification—Combining pre-treatment genetic markers (e.g., *BDNF* rs6265, *GRIN2B* polymorphisms) with circulating miRNAs could help identify patients most likely to benefit from ketamine or ECT.
2. Response monitoring—Longitudinal profiling of miRNAs offers real-time indicators of treatment efficacy, particularly for ECT and ketamine.
3. Drug development—Pathways such as TrkB signaling, NMDA/AMPA receptor balance, and miRNA-regulated immune–neuroplastic crosstalk represent promising therapeutic targets.
4. Cross-treatment biomarkers—The overlap of DNA and miRNA predictors across ketamine, ECT, and TMS suggests that unified biomarker panels may guide multimodal treatment strategies.

### 4.5. Limitations and Future Directions

Despite encouraging findings, current research faces several challenges. Many studies are constrained by small sample sizes, heterogeneous cohorts, and variable treatment protocols, which limit reproducibility. Moreover, methodological heterogeneity in genetic sequencing and miRNA quantification complicates comparability. Most studies remain cross-sectional, leaving the exploration of longitudinal biomarker trajectories unexplored.

Future research should prioritize multi-center replication, standardization of biomarker pipelines, and integration of DNA and RNA markers with neuroimaging and clinical predictors. Longitudinal designs will be crucial for capturing biomarkers relevant to both the acute response and sustained remission.

## 5. Conclusions

Treatment-resistant depression is increasingly understood as a disorder shaped by convergent genetic and epigenetic mechanisms that regulate synaptic plasticity, neurotransmission, and immune–neurobiological cross-talk. Recent evidence highlights consistent associations between variants in *BDNF*, *NTRK2*, *GRIN2A/2B*, *SYN1*, *PTEN*, and *MAPK1* with antidepressant outcomes, implicating neuroplasticity and glutamatergic signaling as central pathways. Complementing this, microRNAs such as miR-1202, miR-16, miR-135, miR-124, miR-223, and miR-146a have emerged as dynamic circulating biomarkers that both reflect and potentially modulate treatment response, particularly in the context of ketamine and electroconvulsive therapy. Together, these findings underscore that a single locus or molecular signal cannot explain TRD; instead, outcomes are determined by the interaction of multiple DNA- and RNA-based regulatory systems. From a translational standpoint, integrating candidate gene variants, GWAS findings, and miRNA profiles offers a promising route toward precision psychiatry, facilitating pre-treatment stratification, real-time monitoring, and mechanism-based drug development. Future studies should prioritize multi-center replication, harmonized methodologies, and longitudinal designs to validate these biomarkers across diverse populations. Incorporating DNA and RNA signatures into clinical care could shift TRD management away from empirical prescribing and toward biologically informed, patient-tailored interventions.

## Figures and Tables

**Table 1:** Summary of included studies.

Reference	Full Title	Methodology/Biomarker Domain
Cai et al. (2024) [33]	miRNAs in treatment-resistant depression: a systematic review	Systematic review; Epigenetics (microRNAs)
Cătană et al. (2025) [60]	MicroRNAs: a novel approach for monitoring treatment response in major depressive disorder?	Review; Epigenetics (microRNAs)
Chen et al. (2021) [47]	Treatment response to low-dose ketamine infusion for treatment-resistant depression: a gene-based genome-wide association study	Candidate gene-based GWAS in TRD; Genetics
Franklin et al. (2025) [64]	Genetics of Response to ECT, TMS, Ketamine and Esketamine	Systematic review of candidate genes, GWAS, and PRS; Genetics
Galbiati et al. (2025) [62]	Plasma microRNA levels after electroconvulsive therapy in treatment-resistant depressed patients	Clinical plasma miRNA profiling; Epigenetics
Kang et al. (2025) [65]	Genetic predictors of ketamine/esketamine response in treatment-resistant depression	Pharmacogenomic association study; Genetics
Kaurani et al. (2023) [61]	MicroRNA modulation after electroconvulsive therapy: markers of response in treatment-resistant depression	Clinical study of plasma miRNAs pre/post ECT; Epigenetics
Paolini et al. (2023) [45]	Association between *NTRK2* polymorphisms, hippocampal volumes and treatment resistance in major depressive disorder	Neuroimaging–genetic study (3T MRI + genotyping); Genetics
Santos et al. (2023) [44]	BDNF, NTRK2, NGFR, CREB1, GSK3B, AKT, MAPK1, MTOR, PTEN, ARC, and SYN1 genetic polymorphisms in antidepressant treatment response phenotypes	Candidate gene analysis in MDD/TRD; Genetics
Saez et al. (2022) [46]	Genetic variables of the glutamatergic system associated with treatment-resistant depression: a review of the literature	Narrative/systematic review; Genetics (NMDA/AMPA pathways)
Statharakos et al. (2023) [63]	Towards precision ECT: a systematic review of epigenetic biomarkers in treatment-resistant depression	Systematic review; Epigenetics
Zelada et al. (2025) [48]	Genetics of response to electroconvulsive therapy, TMS, ketamine and esketamine: insights from the Gen-ECT-ic consortium	Multi-center, multi-omic integration; machine learning; Genetics (PRS/consortium)

**Table 2:** Genetic Predictors of TRD.

Author (Year)	Sample/Methodology	Key Findings
Santos et al. (2023) [44]	80 MDD patients, Texas Medication Algorithm; candidate gene analysis	TRD risk: *PTEN* rs12569998, *SYN1* rs1142636, *BDNF* rs6265. Relapse: *MAPK1* rs6928 (protective), *GSK3B* rs6438552 (higher relapse risk). Pathways: synaptic transmission, glutamatergic signaling.
Saez et al. (2022) [46]	Systematic review of glutamatergic genetics	*GRIN2B* polymorphisms (rs1805502, rs1806201, rs890) linked to TRD, suicidality, low ACC glutamate. *GRIN2A* rs16966731 linked to ketamine response. *GRIA2/GRIA3* variants linked to MDD onset and suicidal ideation.
Paolini et al. (2023) [45]	121 MDD inpatients; 3T MRI + genotyping	*NTRK2* rs1948308 heterozygotes → smaller hippocampal volumes, higher TRD risk. Effect partly mediated by hippocampal volume. No *BDNF* associations (including Val66Met).
Chen et al. (2021) [47]	65 TRD patients, low-dose ketamine; candidate gene-based GWAS	Predictors: *BDNF* rs2049048, *NTRK2* variants (rs10217777, rs10868590, rs77918527). *GRIN2A*, *GRIN2B*, *GRIN2C*, *GRIN3A* linked to rapid/sustained response. *GRIN2A/2B* variants associated with ketamine/norketamine levels.
Kang et al. (2025) [65]	TRD patients treated with ketamine/esketamine; pharmacogenomic analysis	Novel associations: *SYNGR1*, *VAMP2* (synaptic vesicle trafficking); *IL6R*, *TNFAIP3* (immune regulation). Pathways: synapse organization, cytokine signaling. Gene–gene interactions: inflammatory variants modulate ketamine efficacy.
Zelada et al. (2025) [48]	Multi-center; genomic, transcriptomic, proteomic, clinical data; machine learning	Composite panels (*BDNF*, *NTRK2*, *GRIN2A/2B*, *IL6*, *TNFAIP3*) → AUC > 0.80. Combined glutamatergic + immune loci improved prediction of ketamine response.
Franklin et al. (2025) [64]	Systematic review of 34 candidate gene studies and 9 GWAS across ECT, TMS, ketamine, and esketamine	No single variant consistently predicted outcomes. *BDNF* and *COMT* findings were mixed; GWAS remain underpowered but point to glutamatergic and immune processes. Registry-based studies showed depression PRS predicted poorer ECT response, while bipolar PRS predicted better response. Polygenic and integrative approaches show greatest promise.

**Table 3:** Epigenetic regulation in TRD.

Author (Year)	Sample/Methodology	Key Findings
Cătană et al. (2025) [60]	Review of blood-based miRNA biomarkers	Highlighted miR-30a, miR-133b, miR-16, let-7 family; drug-specific modulation (miR-1202 with citalopram, miR-146a-5p with duloxetine); normalization after effective treatment.
Kaurani et al. (2023) [61]	ECT patients, miRNA profiling	Differential regulation of miR-146a, miR-223, miR-126; linked to immune modulation and neuronal plasticity; proposed as ECT-response biomarkers.
Galbiati et al. (2025) [62]	Plasma miRNA in TRD patients before/after ECT	Responders showed ↓miR-223-3p, ↓miR-146a-5p; non-responders showed no change; supports use as response-tracking markers.
Cai et al. (2024) [33]	Systematic review of MDD/TRD miRNAs	Consistent evidence for miR-1202, miR-16, miR-135, miR-124, miR-146a; central roles in plasticity, serotonergic signaling, and neuroinflammation.
Statharakos et al. (2023) [63]	Review of ECT and ketamine miRNA studies	Overlap in regulation of miR-29 family, miR-132, miR-212; both ECT and ketamine modulated miR-29a/c; linked to neuroprotection and plasticity; preliminary evidence for cross-modality biomarkers.

## Data Availability

No new data were created or analyzed in this study.

## Abbreviations

The following abbreviations are used in this manuscript:

ACC	Anterior cingulate cortex
AKT	Protein kinase B
AMPA	α-amino-3-hydroxy-5-methyl-4-isoxazolepropionic acid receptor
ARC	Activity-regulated cytoskeleton-associated protein
BDNF	Brain-derived neurotrophic factor
CaM	Calmodulin
CaMK	Calcium/calmodulin-dependent protein kinase
CACNA1C	Calcium voltage-gated channel subunit alpha1 C
CLOCK	Circadian locomotor output cycles kaput
COMT	Catechol-O-methyltransferase
CREB	cAMP response element-binding protein
DHPS	Deoxyhypusine synthase
DOHH	Deoxyhypusine hydroxylase
ECT	Electroconvulsive therapy
eEF2	Eukaryotic elongation factor 2
eEF2K	Eukaryotic elongation factor 2 kinase
eIF5A	Eukaryotic initiation factor 5A
ERK	Extracellular signal-regulated kinase
FKBP5	FK506 binding protein 5
FT3	Free triiodothyronine
GSK3B	Glycogen synthase kinase 3 beta
GRIA2/3	Glutamate ionotropic AMPA receptor subunit 2/3
GRIN2A/2B	Glutamate ionotropic NMDA receptor subunit 2A/2B
GWAS	Genome-wide association study
HPA	Hypothalamic–pituitary–adrenal axis
IL6	Interleukin 6
IL6R	Interleukin 6 receptor
IP3	Inositol 1,4,5-trisphosphate
JNK	c-Jun N-terminal kinase
lncRNA	Long non-coding RNA
MAPK	Mitogen-activated protein kinase
MAPK1	Mitogen-activated protein kinase 1
MEK	Mitogen-activated protein kinase kinase
miRNA	microRNA
MKP-1	Mitogen-activated protein kinase phosphatase-1
mTOR	Mechanistic target of rapamycin
NMDAR	N-methyl-D-aspartate receptor
NR3C1	Nuclear receptor subfamily 3 group C member 1 (glucocorticoid receptor)
NTRK2 (TrkB)	Neurotrophic receptor tyrosine kinase 2 (tropomyosin receptor kinase B)
PDK1	3-phosphoinositide-dependent protein kinase-1
PI3K	Phosphoinositide 3-kinase
PLC	Phospholipase C
PTEN	Phosphatase and tensin homolog
RAF	Rapidly accelerated fibrosarcoma kinase
RAS	Ras GTPase protein
REDD1	Regulated in development and DNA damage response 1
RHEB	Ras homolog enriched in brain
SK6	Ribosomal protein S6 kinase beta-1
SYN1	Synapsin I
TNFAIP3	Tumor necrosis factor alpha-induced protein 3
TMS	Transcranial magnetic stimulation
TSC	Tuberous sclerosis complex
TRD	Treatment-resistant depression
VAMP2	Vesicle associated membrane protein 2
